# Diacerein and myo-inositol alleviate letrozole-induced PCOS via modulation of HMGB1, SIRT1, and NF-kB: A comparative study

**DOI:** 10.1007/s00210-024-03497-7

**Published:** 2024-10-21

**Authors:** Suzan A. Khodir, Eman Sweed, Shaimaa Mohamed Motawea, Marwa A. Al-Gholam, Sherin Sobhy Elnaidany, Mohamed Zakaria Sayer Dayer, Omnia Ameen

**Affiliations:** 1 Medical Physiology Department, Faculty of Medicine, Menoufia University, Menoufia, 32511 Egypt; 2 Clinical Pharmacology Department, Faculty of Medicine, Menoufia University, Menoufia, 32511 Egypt; 3 Quality Assurance Unit, Menoufia National University, Menoufia, Egypt; 4 Human Anatomy and Embryology Department, Faculty of Medicine, Menoufia University, Menoufia, 32511 Egypt; 5 Medical Biochemistry and Molecular Biology Department, Faculty of Medicine, Menoufia University, Menoufia, 32511 Egypt; 6 Obstetrics &Gynecology, Faculty of Medicine, Menoufia University, Menoufia, 32511 Egypt; 7 Medical Physiology, Menoufia National University, Menoufia, Egypt

**Keywords:** Diacerein, HMGB1, Myo-inositol, NF-kB, PCOS, SIRT 1

## Abstract

Polycystic ovary syndrome (PCOS) is the most prevalent cause of anovulatory infertility in women. Myo-inositol supplementation has displayed effectiveness in curing PCOS patients. Diacerein, an anti-inflammatory medication, has not been extensively studied in the context of reproductive disorders. This study aimed to compare the role of myo-inositol and diacerein in PCOS and the probable mechanisms mediating their actions. Forty adult female rats were divided equally into the following: control, PCOS, PCOS+Myo-inositol, and PCOS+Diacerein groups. Rats were subjected to arterial blood pressure (ABP), electromyography (EMG), and uterine reactivity measurements. Blood samples were collected for measuring hormonal assays, glycemic state, lipid profile, oxidative stress, and inflammatory markers. Ovaries and uteri were extracted for histological examination, including hematoxylin and eosin staining, Masson’s trichrome staining, immunohistochemistry, and rt-PCR analysis of ovarian tissues. PCOS was associated with significant increases in ABP, uterine frequency and amplitude of contraction, luteinizing hormone, testosterone, lipid, glycemic and inflammatory markers, malondialdehyde, high-mobility group box 1 (HMGB1), nuclear factor kappa (NF-kB), ovarian fibrosis, and endometrial thickening. In contrast, there was a significant reduction in follicular stimulating hormone, reduced glutathione, and Sirtuin 1 (SIRT1) when compared with control group. Both myo-inositol and diacerein counteract PCOS changes; but diacerein’s effects were superior to myo-inositol’s for all parameters, except for lipid and glycemic markers. Diacerein possessed anti-inflammatory properties and showed significant efficacy in mitigating the endocrinal, metabolic, and ovarian structural alterations linked to PCOS. Its beneficial actions likely stem from reducing oxidative stress, dyslipidemia, and hyperglycemia, potentially through the modulation of HMGB1, SIRT1, and NF-kB pathways.

## Introduction

Polycystic ovary syndrome (PCOS) is a multifaced endocrinopathy that affects 6–20% of females during reproductive age (Deswal et al. 2020). Its clinical presentations demonstrated diversity in the phenotype of the patients, including reproductive, endocrine, or metabolic features that were either less or more dominant (Joksimovic Jovic et al. 2021).

PCOS is a prevalent endocrine and reproductive disorder characterized by hyperandrogenism, polycystic ovarian morphology, and ovulatory dysfunction, affecting approximately 6% to 10% of the female population (Guney et al. 2022). This condition is also associated with a spectrum of metabolic disturbances, including atherosclerosis, insulin resistance, obesity, dyslipidemia, and an elevated risk of cardiovascular disease (Tokmak et al. 2017). Although the precise pathogenesis of PCOS remains elusive, accumulating evidence implicates genetic factors, adipokines—regulatory proteins secreted by adipose tissue—as well as various inflammatory and anti-inflammatory mediators, as potential contributors to the development and progression of PCOS (Camili et al. 2024).

Insulin resistance (IR), a significant pathological feature in PCOS women, may potentially worsen the symptoms associated with this condition. While the precise mechanisms responsible for IR in PCOS are not fully understood, there is a strong correlation between PCOS and hyperinsulinemia, resulting in anovulation and impaired follicular development (Purwar and Nagpure 2022).

A significant portion (67%) of patients with PCOS achieves spontaneous pregnancies. However, the remaining individuals often require assisted reproductive techniques or ovulation induction to conceive (Hudecova et al. 2009)***.*** However, in these pregnancies, there were high occurrences of preterm birth, ectopic pregnancy, and spontaneous abortion, indicating a correlation between these pregnancy complications and hyperandrogenism, hyperinsulinemia, IR, and endometrial changes (Si et al. 2023)***.*** Moreover, PCOS patients often experience infertility, early pregnancy losses, and subfertility due to disruptions in the hypothalamic-pituitary-ovarian axis. Nevertheless, the available data regarding the correlation between these clinical conditions that might arise in PCOS and the potential functional and molecular alternations in the uterus myometrium is limited (Yusuf et al. 2023)***.***

Sirtuin, a group of NAD+-dependent deacetylases, acts as metabolic receptors, regulating homeostasis in the human body which are triggered during calorie restriction (Li et al. 2023). SIRT1 is involved in the pathophysiological processes of oxidative stress, autophagy, ovulation disruption, and IR, which may be a crucial connection in the incidence of PCOS (Wu et al. 2022). Reduced SIRT1 activity or impaired signaling is a common pathological feature of cardiovascular diseases, non-alcoholic fatty liver, and other inflammatory and metabolic disorders as these disorders are associated with PCOS. Caloric restriction boosts ovarian reserves, implying that sirtuins could regulate reproductive ability (Li et al. 2023).

High-mobility group box 1 (HMGB1) is an endogenous danger signal that activates the inflammatory response and aids in the control of tissue repair and inflammatory responses. Currently, it is acknowledged as a proinflammatory cytokine (Al-kuraishy et al. 2022)***.*** HMGB1 inflammatory function has been demonstrated to be significant in the pathogenesis of numerous diseases like type 2 diabetes (T2DM) (Yang et al. 2023). It has been detected in the blood of diabetic individuals. Moreover, the suppression of HMGB1 has been demonstrated to contribute to the alleviation of IR. Prior research has documented the existence of mild inflammation and elevated levels of HMGB1 in both follicular fluid and circulation of PCOS women (Cirillo et al. 2020).

Myoinositol is the predominant isoform of inositol. In its free form, inositol is a type of sugar alcohol that can be found in the diet. It functions as a secondary messenger in the intracellular insulin pathway, enhancing cellular glucose uptake, metabolism, and insulin sensitivity (Antonowski et al. 2019). Myoinositol has become more prevalent in clinical reproductive practice over the last 20 years. It has been found to have a beneficial effect on certain types of tumors and metabolic disorders such as infertility, T2DM, thyroid disorders, and PCOS (Chatree et al. 2020; Gudović et al. 2024)***.***

Diacerein, an anthraquinone derivative, has been used for the management of osteoarthritis (Zeng et al. 2023). Prior studies have proven that diacerein exhibits a diverse array of anti-apoptotic, antioxidant, and anti-inflammatory actions (Leite et al. 2019; Rasheed et al. 2023). Diacerein’s anti-inflammatory properties have been linked to its capacity to hinder the synthesis of inflammatory cytokines like Interleukin 1-β (IL-1β) and tumor necrosis factor-α (TNF-α), as well as to the reduction of IL-1β receptors (Almezgagi et al. 2020)***.*** Furthermore, multiple clinical studies have proven the advantageous impact of diacerein on insulin secretion as well as metabolic control (Tres et al. 2018)***.***

This study aimed to examine and compare the potential effects of diacerein and myoinositol in PCOS produced by letrozole (in adult female rats). In addition, the study aimed to elucidate the underlying mechanisms mediating their actions, with a specific focus on the SIRT1/HMGB1/IL-1β/nuclear factor kappa (NF-κB) signaling pathways. To the best of our knowledge, this is the first study to compare the effects of myo-inositol and diacerein in PCOS.

## Materials and methods

### Experimental animals

Using the statistics and sample size pro program version 6, the smallest sample size is determined to be forty rats. The study has an 80% power and a 95% confidence level. Eight to 10-week-old, healthy, cyclic adult female albino Wistar rats weighing 200–250 gm participated in the current study. Ethical permissions from the Research Ethical Committee at the Faculty of Medicine, Menoufia University, Egypt, with IRB No 12/2023 BIO-6 were obtained. ARRIVE criteria were followed throughout the experimental procedures (Percie du Sert et al. 2020). The rats were kept in cages with wire mesh (80x40x30 cm). During the study period, all rats were provided with regular access to food and water following 2 weeks of acclimatization to a consistent environment with a 12:12-h light/dark cycle.

**Fig. 1:**
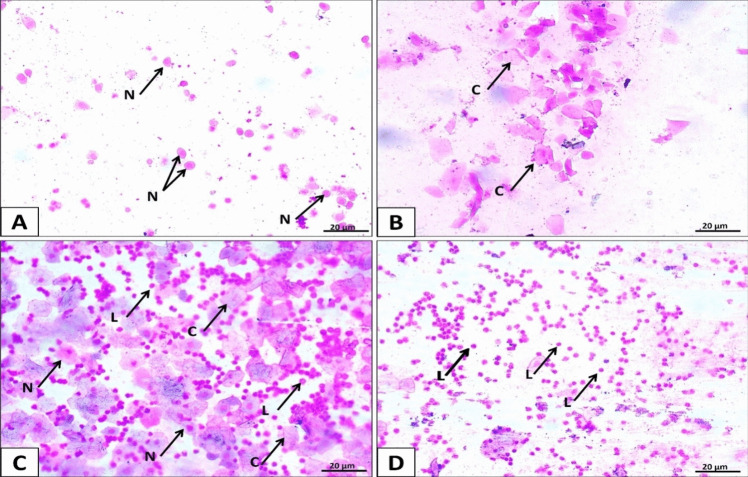
Representing photomicrograph of vaginal smear presents every stage of the rat normal estrous cycle. Vaginal smear images revealed the following cells: nucleated epithelial (N), cornified epithelial (C), and leukocytes (L). Four stages represent the estrous cycle: proestrus (**A**), estrus (**B**), metestrus (**C**), and diestrus (**D**) (Giemsa-stained vaginal smears X400, 20 µm scale bar)

Every rat was examined daily using vaginal smears. Vaginal secretions were collected using a plastic pipette filled with 10 ml of normal saline (NaCl 0.9%). The normal saline was applied to a clean glass slide and left to dry. The slides were stained with Giemsa for 20 min before inspecting the cells under a light microscope. The various phases of the estrus cycle were identified by analyzing the proportion of cells present in the vaginal smear (Ajayi and Akhigbe 2020). Nucleated epithelial cells were prevalent during proestrus (Fig. 1A), estrus phase (Fig. 1B) showed predominance of cornified cells, metestrus phase (Fig. 1C) revealed an equal amount of nucleated epithelial cells, cornified cells, and leucocytes and leucocytes were dominantly present during the diestrus phase (Fig. 1D) in the vaginal smear. The rat estrous cycle usually lasts about 4 days, and only those with regular estrous cycles were used for this study.

### Experimental design

The rats were divided randomly into 4 groups (10/group):

1. Control group: rats were fed with regular chow and received carboxy methyl cellulose (CMC) 2 ml/kg/day via gastric lavage for 7 weeks.
2. PCOS group: induction of PCOS was done by daily administration of Letrozole (Techno Pharco Company, New Borg El Arab City, Alexandria, Egypt) at a dose of 1 mg/kg dissolved in 0.5% CMC via gastric lavage for 3 weeks (Ibrahim et al. 2022).
3. PCOS+Myo-inositol group: induction of PCOS was done as in PCOS group, followed by daily administration of myo-inositol (Fairhaven Health Company, Middletown, CT, USA) via gastric gavage at a dose of 50 mg/kg for 4 weeks (Zhang et al. 2020b).
4. PCOS+Diacerein group: induction of PCOS was done as in PCOS group, followed by daily administration of diacerein (Eva Pharma Company, 10th of Ramadan, Cairo, Egypt) via gastric gavage at a dose 50 mg/ kg for 4 weeks (Ibrahim et al. 2022).

All the rats were weighed, and their arterial blood pressure (ABP) was measured after the full 7 weeks had passed. Uterine reactivity and electromyography (EMG) of the myometrium were then assessed. After obtaining blood samples from the retro-orbital venous plexus, rats were sacrificed by dislocating and elongating their cervical spines. The uteri and ovaries were extracted from all rats, and the right ovary was preserved in 10% formalin saline for evaluation using immunohistochemistry and histopathology. Real-time polymerase chain reaction (RT-PCR) was performed on the left ovary tissue.

### Experimental procedures

#### Confirmation of PCOS induction

**Fig. 2:**
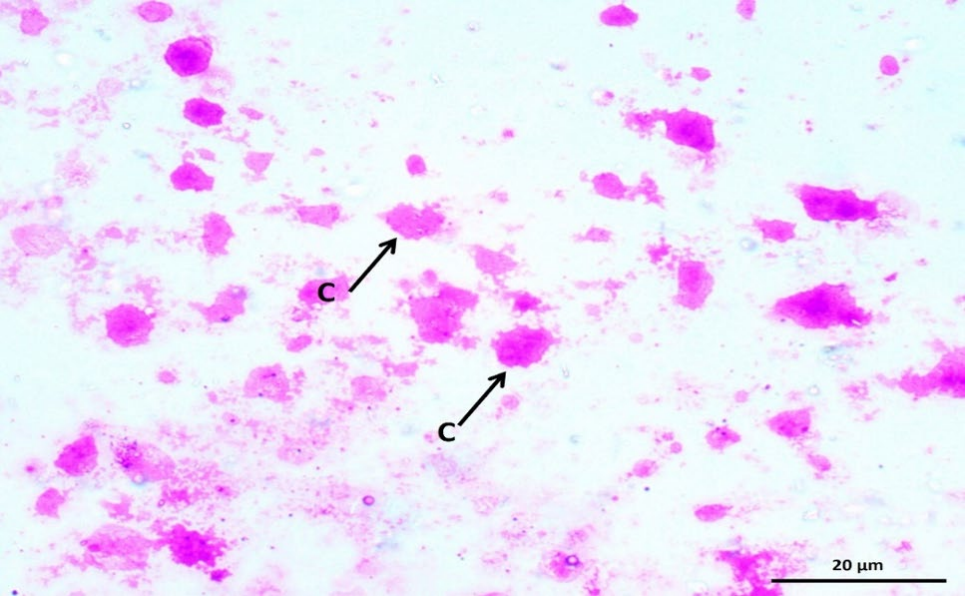
Representing photomicrograph of a vaginal smear from a female rat from the PCOS group showing persistent vaginal cornification (C) (Vaginal smear stained by Giemsa stain X400)

After letrozole was administered for three weeks, the PCOS model was confirmed by measuring fasting blood glucose (FBG), hormonal assay of luteinizing hormone (LH), follicular stimulating hormone (FSH), and testosterone and collecting vaginal smears daily. Disturbed estrus cycles and persistent vaginal cornification (Fig. 2) indicate the presence of ovarian cysts and early confirmation of PCOS induction.

#### Non-invasive blood pressure measurement

MP 36R Ultimate System ® (BIOPAC, Aero Camino, USA) was used to assess the diastolic and systolic blood pressures non-invasively. The rat was placed inside the restrainer and placed in the heating part of the animal heating chamber prior to the experiment. After 30 min, the rat’s temperature reached the desired level. The sensor was attached to the rat’s tail, while the restrainer was still inside the animal heating chamber. Once the rat was relaxed, blood pressure readings were obtained. Acknowledge software was used to analyze numerical data (Das et al. 2020).

#### Detection of myoelectrical activity

Electrical activity of the myometrium was recorded by using MP 36R Ultimate System ® (BIOPAC, Aero Camino, USA). Rats were anaesthetized intraperitoneally with a combination of ketamine (36 mg/kg) and xylazine solution (4 mg/kg). A laparotomy was performed via a midline incision from the symphysis pubis to the xiphoid process, and the total gastrointestinal tract and bladder were resected, and the rat’s uterus was exposed. Two shielded electrodes (EL508S) were used for the signal inputs, and one unshielded electrode (EL508) was used for ground to measure the EMG. The grounding electrode was positioned on the root of the tail, while the active and reference electrodes were inserted into the myometrium separated by a distance of 8 mm. Acknowledge software was used to analyze the mean frequency and mean power numerical data (Ünsal and Özcan 2018).

#### Assessment of uterine reactivity

The uterus was promptly removed, and any attached tissues were cautiously eliminated in a petri dish that contained Krebs solution (composition in mM: Na_2_EDTA 0.01, glucose 11, KH_2_PO_4_ 1.2, NaHCO_3_ 25, MgSO_4_ 1.2, CaCl_2_ 2.5, KCl 4.8, and NaCl 118). Two strips, measuring about one mm in width and 1 cm in length, were obtained from a single rat uterus.

The uterine strips were suspended in organ baths filled with Krebs’ solution, maintained at a temperature of 37 °C, and exposed to 95% O2 under an initial tension of 1 g. The isometric tissue responses were measured utilizing a force transducer (COMMAT-Ankara-Turkey) and displayed on a Biopac acquisition system (Biopac Systems, Goleta-CA-USA). Additionally, the strips were equilibrated at optimal resting tensions (for 60 min), and the bath was replenished every 20 min (with a fresh Krebs solution). After reaching a state of equilibrium, the uterine strips were stimulated to contract utilizing acetylcholine (Ach) (10^−6^-10^-2^ M) or oxytocin (OXY) (10^−6^-10^-2^ M). The contractions were measured in gram force. All medications and substances were obtained from Sigma (Sigma Chemical Company-St. Louis-MO-USA) (Aktas et al. 2019).

### Biochemical measurements

#### Blood sampling

Using a non-heparinized capillary tube, fasting blood samples were drawn from the retro-orbital venous plexus. A 4 ml of blood were taken, allowed to coagulate for 10 min at room temperature in a water bath, and then centrifuged for 10 min at a speed of 4000 rotations per minute (r.p.m.). The serum was kept frozen at − 20 °C until it was required for further examination.

#### Calculation of HOMA-IR index

HOMA-IR index = [fasting serum insulin (µU/ml) X fasting serum glucose (mg/dl)]/405 (Khodir et al. 2020).

#### Biochemical analysis

Serum samples were used for estimation of glucose (Diamond Diagnostic, Egypt) and insulin (DRG Instruments GmbH, Germany). ELISA kits for LH (Elabscience Biotechnology Inc. Houston, Texas, USA), FSH and testosterone (MyBioSource, San Diego, CA, USA), IL-1β (Abcam, Cambridge, UK), and TNF-α (MyBioSource, San Diego, CA, USA) were used. Malondialdehyde (MDA), reduced glutathione (GSH), fasting serum cholesterol, and triglyceride (TG) concentrations were determined using colorimetric kits (Biodiagnostic Company, Dokki, Giza, Egypt). Assays were performed according to the manufacturers’ instructions.

### Molecular measurements

#### Quantitative assay of hmgb1 and sirt1 gene expression using rt-PCR

A quantitative analysis of HMGB1 and SIRT1 gene expression was conducted utilizing rt-PCR. Ovarian tissues were prepared for total RNA isolation using Universal Kit and Qiagen RN Easy (USA). Subsequently, the RNA’s quality and purity were assessed, and RNA was stored at a temperature of – 80 °C. First, cDNA was synthesized using QuantiTect Reverse Transcription Kit (USA) and Applied Biosystems 2720 thermal cycler (Singapore) for a single cycle. The rt-PCR reaction utilized GAPDH primers as a control for RNA loading. The second phase entailed cDNA amplification. For Relative Quantification (RQ) of HMGB1 and SIRT 1 gene expression, cDNA was utilized in SYBR green-based quantitative rt-PCR with the SensiFASTTM SYBR Low-ROX Kit (USA) and the following designed primers (Midland, Texas):

The forward primer for HMGB 1 was *(TGAGGGACAAAAGCCACTC),* and the reverse primer was *(TTGGGAGGGCGGAGAATC*).

The forward primer for SIRT1 was *(AGA AACAATTCCTCCACCTGA)*, and the reverse primer was *(GCTTTGGTGGTTCTGAAAGG*).

The forward primer for GAPDH was (*TGCACCACCAACTGCTTAGC)*, and the reverse primer was (*GGCATGGACTGTGGTCATGAG*). The PCR reaction was carried out in a total volume of 20 μL, consisting of 10 μL SYBR Green, 5 μL RNase-free water, 3 μL cDNA, 1 μL forward primer, and 1 μL reverse primer. The cycling conditions included an initial denaturation for 50 cycles at 94°C for 30 seconds, followed by annealing at 55°C for 30 seconds, and extension at 72°C for 33 seconds. The data analysis was conducted utilizing the 2.0.1 iteration of the Applied Biosystems 7500 software. The expression of the HMGB 1 and SIRT 1 genes was assessed using the comparative ∆∆Ct method. This method involves normalizing the mRNA content of the target gene to a control and an endogenous reference gene (GAPDH).

### Histopathological examinations

#### Histological study

The ovaries and uterus were preserved in 10% neutral formaldehyde. The tissues then were prepared for Paraffin sections of approximately 5-μm thickness. The sections were taken and stained with hematoxylin and eosin to analyze the histological alterations of the ovarian and uterine tissues and with Masson trichrome to detect collagen fibers (Bancroft and Layton 2018).

#### Immunohistochemical study

Immunohistochemical staining of ovarian tissues was done in all studied groups for detection of nuclear factor κB (NF-κB). Briefly, deparaffinized sections were hydrated in graded ethanol (100%, 95%, and 80%) for 1 min and then washed with water. The tissues were treated with 10 mM Citrate Buffer pH 6.0 for antigen retrieval in an oven for 15 min and then chilled for 20 min before being washed. H2O2 (3%) was utilized for 10 min to stop endogenous peroxidase activity, and the tissues were rinsed with distilled H2O. The slides were treated overnight with primary antibodies rabbit anti NF-κB polyclonal antibody at a dilution of 1:100 at 4 °C and then rinsed with phosphate buffered saline. The tissues were treated with poly horseradish peroxidase conjugate for 30 min at room temperature and then rinsed with phosphate buffered saline. The tissues were treated with 3,3′ diaminobenzidine chromogen to evaluate staining progress. After that, slides were cleaned, counterstained with hematoxylin, dehydrated, clarified, and then cover-slipped to be imaged with an Olympus light microscope (Kiernan 2015).

### Quantitative analysis

Five non-overlapping fields per section were randomly captured by a Leica Microscope DML B2/11888111 equipped with a camera DFC450 for histological and immunohistochemical quantitative assessment. Image J software (MD, USA) was used to measure collagen fiber, immunoreactivity (400X), and thickness of the endometrium and myometrium (200X), in at least five fields from each animal, and averaged per field for each animal.

### Statistical analysis

Shapiro–Wilk test was used to assess the normality of the data distribution. Results are expressed as mean ± standard deviation (SD). Analysis of variances (ANOVA) and 2-way ANOVA were used for statistical analysis of the different groups followed by post hoc Tukey’s test, using GraphPad Prism software version 9.0.2 (GraphPad Software, Boston, Massachusetts, USA) and SPSS version 26 (SPSS Inc., Armonk, NY, USA). *P* values < 0.05 were considered significant.

## Results

### Blood pressure

**Fig. 3:**
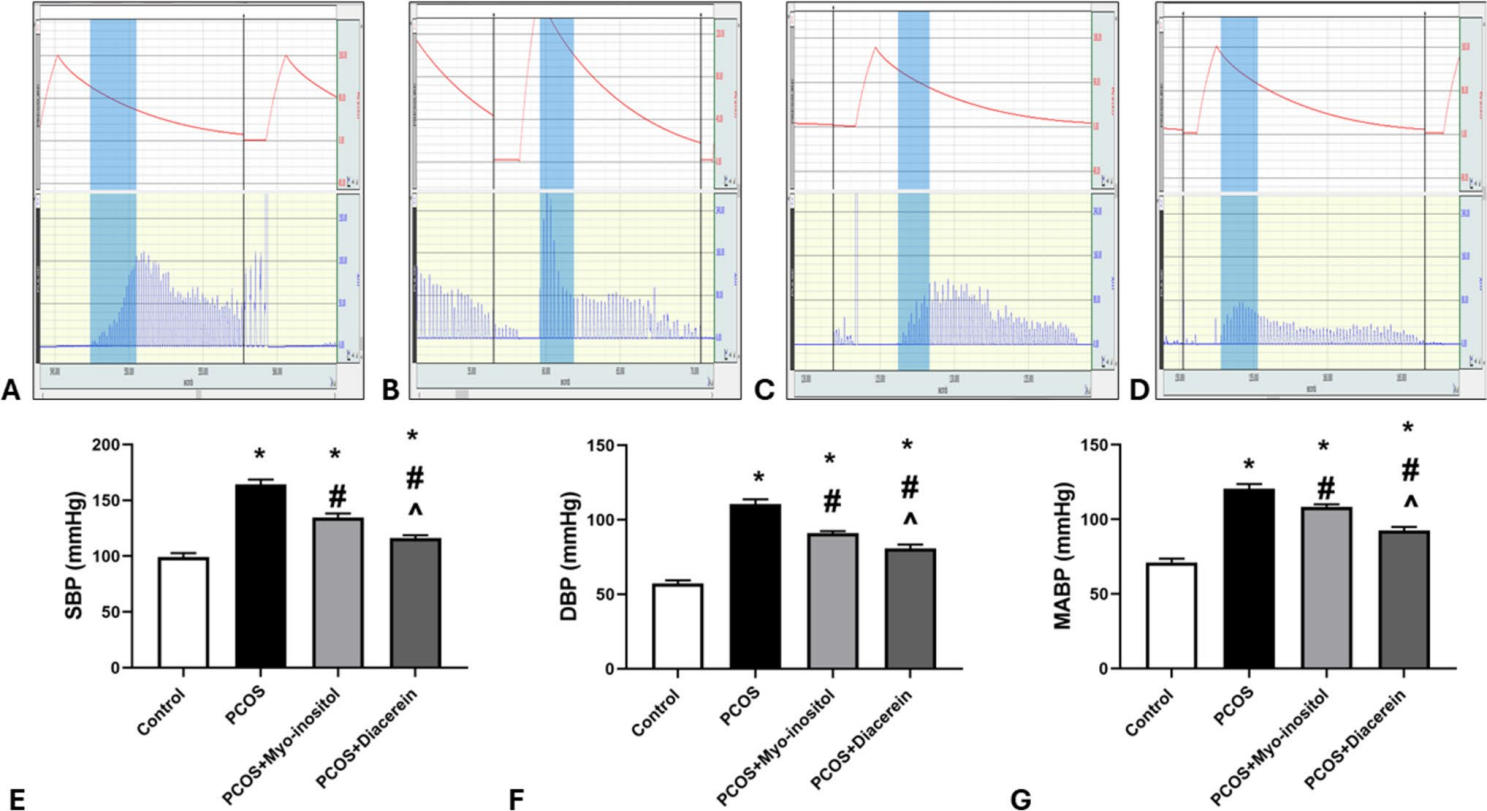
Effects of Myo-inositol and diacerein on systolic blood pressure (SBP), diastolic blood pressure (DBP), and mean arterial blood pressure (MABP) levels in PCOS. **A** control, **B** PCOS, **C** PCOS+Myo-inositol, **D** PCOS+Diacerein, **E** SBP, **F** DBP, and **G** MABP. Compared using the ANOVA test; *p < 0.05 compared with control, #*p* < 0.05 compared with PCOS and ^ compared with PCOS+Myo-inositol. PCOS, polycystic ovary syndrome

The mean values of systolic blood pressure (SBP), diastolic blood pressure (DBP), and mean arterial blood pressure (MABP) levels in PCOSgroup were significantly higher than that in control group (164.33 ± 4.17 vs 99 ± 3.74, 110.6 ± 3.26 vs 57.33 ± 2.06, 120.7 ± 2.96 vs 71.16 ± 2.54, mmHg respectively; *P* < 0.05). SBP, DBP, and MABP levels in PCOS+Myo-inositol (134.5 ± 3.7, 91 ± 1.4, 108.4 ± 1.7 mmHg respectively) and PCOS+Diacerein (116.3 ± 2.5, 80.8 ± 2.6, 92.6 ± 2.3 mmHg respectively) groups were significantly lower than that in PCOSgroup but still significantly higher than that in control (*P* < 0.05). SBP, DBP, and MABP levels in PCOS+Myo-inositol were significantly higher (*P* < 0.05) than that of PCOS+Diacerein group (Fig. 3A, B, C, D, E, F, and G).

### EMG

**Fig. 4:**
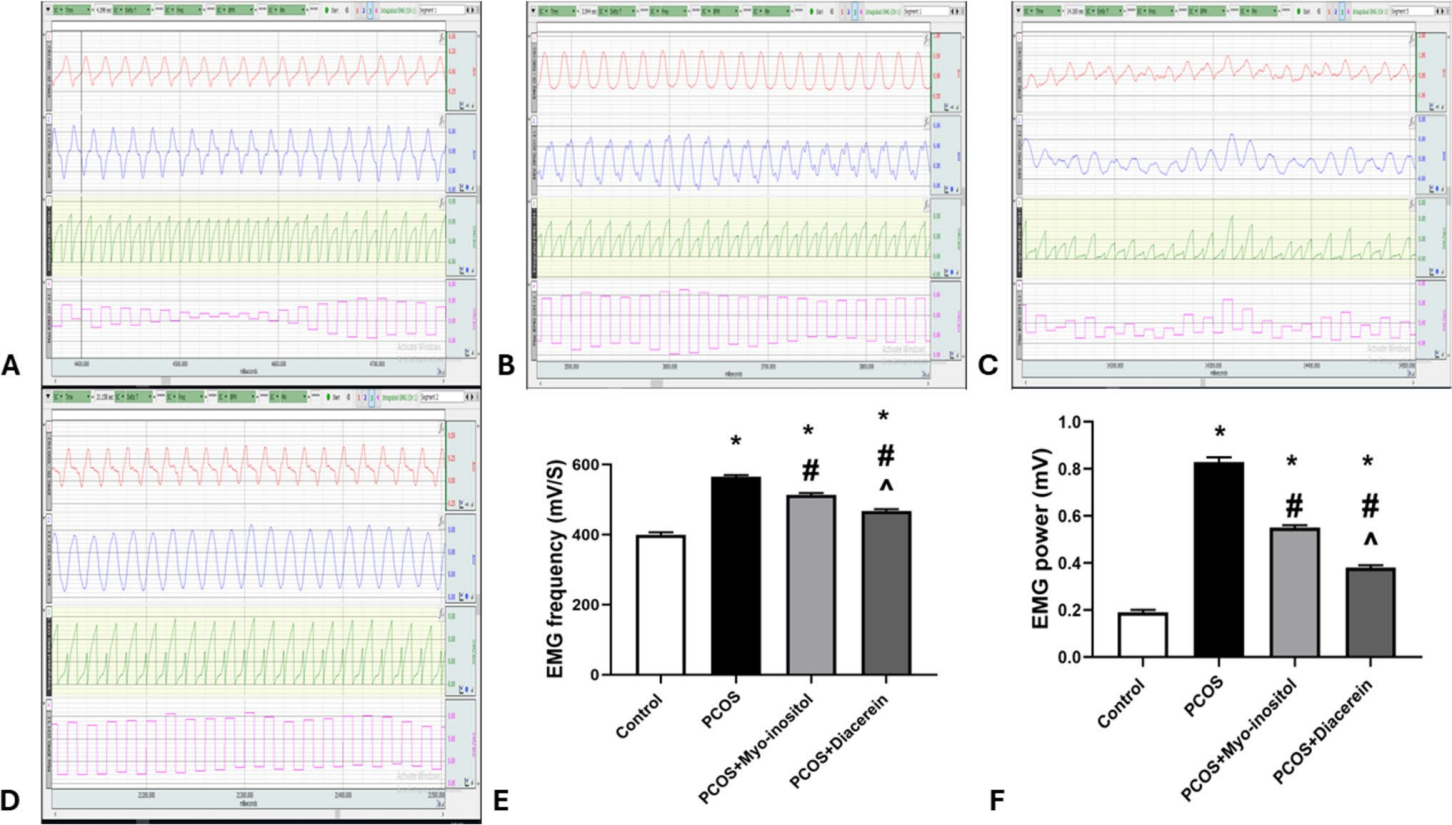
Effects of Myo-inositol and diacerein on electromyogram (EMG). **A** control, **B** PCOS, **C** PCOS+Myo-inositol, **D** PCOS+Diacerein, **E** EMG frequency (mV/S), and **F** EMG power (mv). Compared using the ANOVA test; *p < 0.05 compared with control, #*p* < 0.05 compared with PCOS, and ^ compared with PCOS+Myo-inositol. PCOS, polycystic ovary syndrome

The mean EMG frequency and power levels in PCOS group were significantly higher than that in control group (565.5 ± 4.63 vs 399.5 ± 7.12 mV/S and 0.83 ± 0.02 vs 0.19 ± 0.01 mV respectively; *P* < 0.05). The mean EMG frequency and power levels in PCOS+Myo-inositol (513 ± 5.65 mV/S and 0.55 ± 0.011 mV) and PCOS+Diacerein (467.5 ± 5 mV/S and 0.385 ± 0.01 mV) groups were significantly lower than that in PCOS group but still significantly higher than that in control (*P* < 0.05). The mean EMG frequency and power levels in PCOS+Myo-inositol were significantly higher (*P* < 0.05) than that of PCOS+Diacerein group (Fig. 4A, B, C, D, E, and F).

### Uterine reactivity

Exposing the isolated uterus to Ach and OXY resulted in significantly higher amplitude and frequency of rhythmic contractions in the PCOS group compared with the control group (*P* < 0.05).

**Fig. 5:**
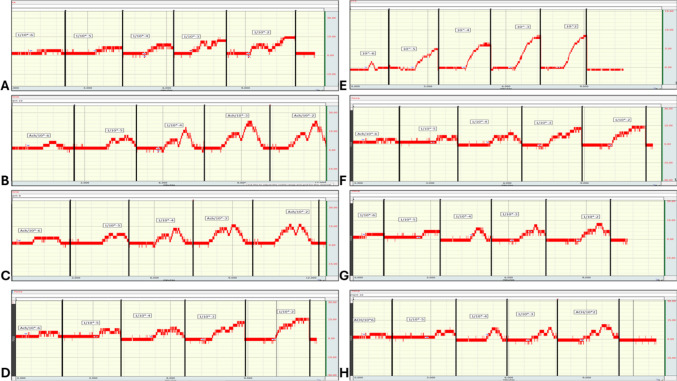
Effects of Ach and OXY on uterine reactivity in different groups. **A** and **E** control, **B** and **F** PCOS, **C** and **G** PCOS+Myo-inositol, **D** and **H** PCOS+Diacerein. Compared using the two-way ANOVA test; *p < 0.05 compared with control, #*p* < 0.05 compared with PCOS, and ^ compared with PCOS+Myo-inositol. PCOS, polycystic ovary syndrome

While in PCOS+Myo-inositol and PCOS+Diacerein, there was decrease in amplitude and frequency of rhythmic contractions after exposure of isolated uterus to Ach and OXY (Table 1; Fig. 5A, B, C, D, E, F, G, and H).

**Table 1:** Effects of Myo-inositol and diacerein on uterine reactivity

	Control	PCOS	PCOS+Myo-Inositol	PCOS+Diacerein
OXY amplitude				
10^-6^	2.82±0.80	4.95±0.34 *	4.09±0.16 *^#^	3.25±0.37 ^#^^
10^-5^	4.95±0.34	7.42±0.47 *	6.74±0.27 *^#^	6.24±0.076 *^#^
10^-4^	7.42±0.47	9.5±0.45 *	8.79±0.62 *^#^	8.14±0.051 *^#^^
10^-3^	9.5±0.45	14.6±0.40 *	11.97±0.71 *^#^	10.7±0.8 *^#^^
10^-2^	9.5±0.45	15±0.55 *	12.2±0.32 *^#^	11.7±0.31 *^#^
Ach amplitude				
10^-6^	4.56±1.08	7.25±1.56 *	6.85±1.45 *	5.9±0.52 *^#^^
10^-5^	6.5±0.22	11.97±0.71 *	9.5±0.45 *^#^	8.43±0.50 *^#^^
10^-4^	9.37±0.49	21.9±0.91 *	13±0.48 *^#^	12.2±0.32 *^#^
10^-3^	13.3±0.79	26.9±0.3 *	16.8±0.7 *^#^	14.6±0.40 *^#^^
10^-2^	15.60±1.2	26.9±0.4 *	17.5±0.90 *^#^	16±0.35 ^#^^
Ach frequency				
10^-6^	11.97±0.71	13.3±0.79 *	13±0.48 *	12.4±0.35 ^#^
10^-5^	12.2±0.32	13.8±0.67 *	13.2±0.7 *	12.6±0.64 ^#^
10^-4^	14.2±0.33	15±0.55 *	14.6±0.40	14.33±0.32
10^-3^	15.3±0.56	18.5±0.58 *	17±0.404 *^#^	15.602±1.29 ^#^^
10^-2^	16±0.35	19.6±0.54 *	17.5±0.90 *^#^	17±0.40 *^#^
OXY frequency				
10^-6^	11.7±0.35	12.5±0.47 *	12.2±0.32	11.97±0.71
10^-5^	12.9±0.43	13.8±0.67 *	13.3±0.79	13±0.48 ^#^
10^-4^	14.2±0.33	15.58±0.56 *	15.3±0.56 *	14.6±0.40 ^#^
10^-3^	16±0.35	20.2±4.11 *	18±0.70 *^#^	17±0.40 *^#^^
10^-2^	16.8±0.7	23.2±1.4 *	19.6±0.54 *^#^	18.5±0.58 *^#^^

Compared using the two-way ANOVA test; *p < 0.05 compared with control, #*p* < 0.05 compared with PCOS, and ^ compared with PCOS+Myo-inositol. PCOS, polycystic ovary syndrome

### Biochemical results

#### Hormonal assay

**Fig. 6:**
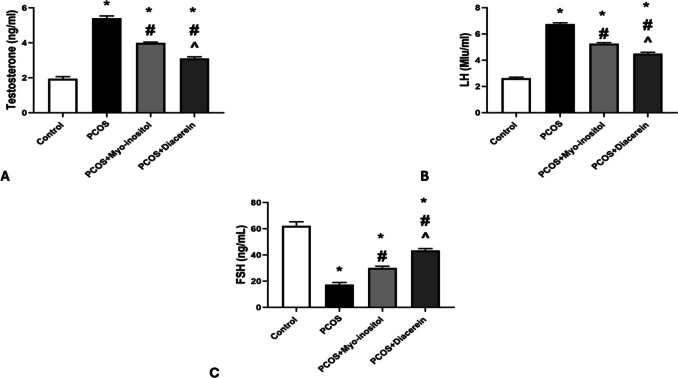
Effects of Myo-inositol and diacerein on hormonal assay. **A** Testosterone, **B** LH, **C** FSH. Compared using the ANOVA test; *p < 0.05 compared with control, #*p* < 0.05 compared with PCOS, and ^ compared with PCOS+Myo-inositol. *PCOS* polycystic ovary syndrome, *LH* luteinizing hormone, *FSH* follicle stimulating hormone

The mean values of testosterone and LH in PCOSgroup were significantly higher than that in control group (5.40 ± 0.14 vs 1.94± 0.13 ng/ml and 6.75 ± 0.10 vs 2.62 ± 0.08 Mlu/ml respectively; *P* < 0.05). Testosterone and LH levels in PCOS + Myo-inositol (3.99 ± 0.05 ng/ml, 5.26 ± 0.08 Mlu/ml) and PCOS+Diacerein (3.10 ± 0.10 ng/ml, 4.49 ± 0.11 Mlu/ml) groups were significantly lower than that in PCOSgroup but still significantly higher than that in control (*P* < 0.05). The testosterone and LH levels in PCOS+Myo-inositol were significantly higher (*P* < 0.05) than that of PCOS+ Diacerein (Fig. 6A and B).

The mean value of FSH in PCOSgroup was significantly lower than that in control group (17.41 ± 1.6 vs 62.16 ± 3.18 ng/ml respectively; *P* < 0.05). FSH levels in PCOS + Myo-inositol (30.06 ± 1.38 ng/ml) and PCOS+Diacerein (43.41 ± 1.46 ng/ml) groups were significantly higher than that in PCOSgroup but still significantly lower than that in control (*P* < 0.05). The FSH levels in PCOS+Myo-inositol were significantly lower (*P* < 0.05) than that of PCOS+ Diacerein (Fig 6C).

#### Glycemic state

**Fig. 7:**
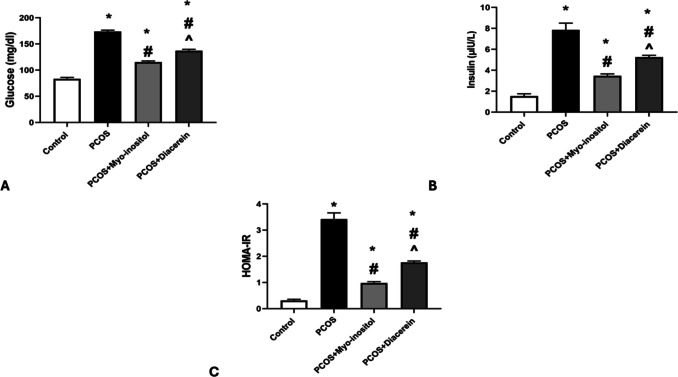
Effects of Myo-inositol and diacerein on glycemic state. **A** Glucose, **B** insulin level, and **C** HOMA-IR. Compared using the ANOVA test; *p < 0.05 compared with control, #*p* < 0.05 compared with PCOS, and ^ compared with PCOS+Myo-inositol. PCOS, polycystic ovary syndrome

The mean values of glucose, insulin level, and HOMA-IR in PCOS were significantly higher than in control group (173.83 ± 2.63 vs 83.50 ± 2.58 mg/dl, 7.86 ± 0.64 vs 1.54 ± 0.21 µIU/L , and 3.42 ± 0.24 vs 0.31 ± 0.05 respectively;* P* < 0.05), while the glucose level, insulin level, and HOMA-IR in PCOS+Myo-inositol (115.50 ± 2.16 mg/dl, 3.47 ± 0.18 µIU/L, and 0.98 ± 0.05 respectively) and PCOS+Diacerein (137.33 ± 2.50 mg/dl, 5.25 ± 0.18 µIU/L, and 1.77 ± 0.05 respectively) groups were significantly lower than that in PCOS group (*P* < 0.05) but still significantly higher than that in control *(P* < 0.05). The glucose level insulin level and HOMA-IR in PCOS+Myo-inositol were significantly lower (*P* < 0.05) than that of PCOS+Diacerein group (Fig. 7A, B, and C).

#### Lipid profile

**Fig. 8:**
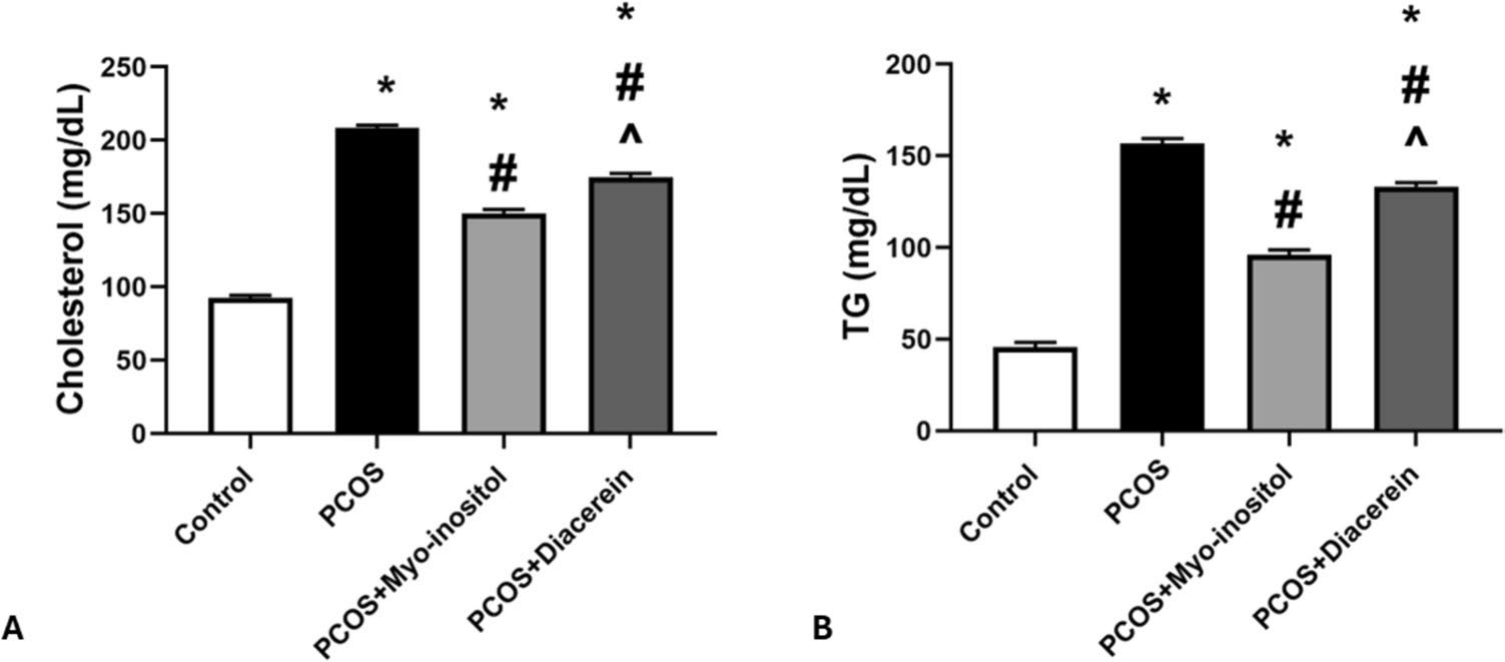
Effects of Myo-inositol and diacerein on lipid profile. **A** Cholesterol, **B** TG. Compared using the ANOVA test; *p < 0.05 compared with control, #*p* < 0.05 compared with PCOS, and ^ compared with PCOS+Myo-inositol. *PCOS*, polycystic ovary syndrome, *TG* triglycerides

The mean values of cholesterol and TG levels in PCOS group were significantly higher than that in control group (208.50 ± 1.87 vs 92.50 ± 1.87 mg/dL and 156.67 ± 2.73 vs 45.83 ± 2.48 mg/dL respectively; *P* < 0.05). Cholesterol and TG levels in PCOS+Myo-inositol (150.17 ± 2.71 mg/dL and 96.16 ± 2.63 mg/dL) and PCOS+Diacerein (174.67 ± 2.80 mg/dL and 133.33 ± 2.16 mg/dL) groups were significantly lower than that in PCOS group but still significantly higher than that in control (*P* < 0.05). Cholesterol and TG levels in PCOS+Myo-inositol were significantly lower (*P* < 0.05) than that of PCOS+Diacerein group (Fig. 8A and B).

#### Oxidative stress markers

**Fig. 9:**
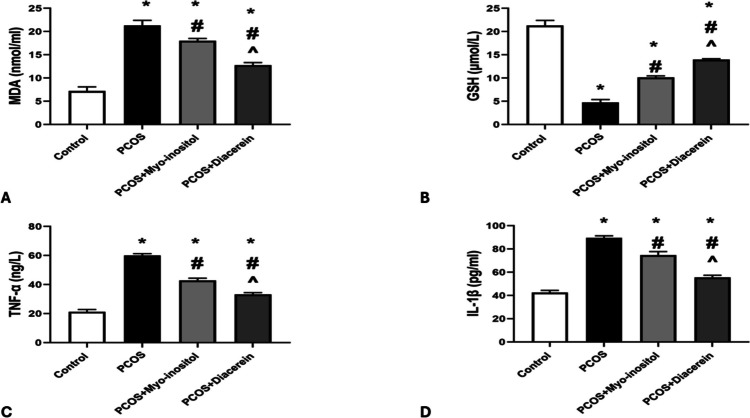
Effects of Myo-inositol and diacerein on oxidative stress markers and inflammatory mediators. **A** MDA, **B** GSH, **C** TNF-α, and **D** IL-1β. Compared using the ANOVA test; *p < 0.05 compared with control, #*p* < 0.05 compared with PCOS, and ^ compared with PCOS+Myo-inositol. PCOS, polycystic ovary syndrome; MDA, malondialdehyde; GSH, glutathione; TNF-α, tumor necrosis factor-alpha; and IL-1β, Interleukin 1-β

The mean value of MDA levels in PCOS group was significantly higher than that in control group (21.33 ± 1.08 vs 7.23 ± 0.86 nmol/ml respectively). The MDA levels in PCOS+Myo-inositol (18.03 ± 0.47 nmol/ml) and PCOS+Diacerein (12.76 ± 0.56 nmol/ml) groups were significantly lower than that in PCOS group but still significantly higher than that in control (*P* < 0.05). MDA values in PCOS+Myo-inositol were significantly higher (*P* < 0.05) than that of PCOS+Diacerein group (Fig. 9A)

The mean value of GSH levels in PCOS group was significantly lower than that in control group (4.75 ± 0.61 vs 21.33 ± 1.08 μmol/L, respectively). The GSH levels in PCOS+Myo-inositol (10.15 ± 0.32 μmol/L) and PCOS+Diacerein (13.98 ± 0.17 μmol/L) groups were significantly lower than that in control group but still significantly higher than that in PCOS (*P* < 0.05). GSH values in PCOS+Myo-inositol were significantly lower (*P* < 0.05) than that of PCOS + Diacerein group (Fig. 9B).

#### Inflammatory mediators

The mean levels of TNF-α and IL-1β in PCOS group were significantly higher than that in control group (60.00 ± 1.30 vs 21.33 ± 1.53 ng/L and 89.50 ±1.87 vs 42.50 ± 1.87 pg/ml respectively, *P* < 0.05). TNF-α levels and IL-1β in PCOS+Myo-inositol (42.93 ± 1.45 ng/L and 74.83 ± 2.92 pg/ml) and PCOS+Diacerein (33.33 ± 1.08 ng/L and 55.50 ± 1.87 pg/ml) groups were significantly lower than that in PCOS group but still significantly higher than that in control group (*P* < 0.05). TNF-α levels and IL-1β in PCOS+Myo-inositol were significantly higher (*P* < 0.05) than that of PCOS+Diacerein group (Fig. 9C, D).

### Molecular results

#### SIRT1 and HMGB1 gene expression

The expression level of SIRT1 gene was significantly down regulated in PCOS (0.38 ± 0.02) than that in control group (1) (*P* < 0.05). SIRT1 gene expression in PCOS+ Myo-inositol (0.59 ± 0.01) and PCOS+Diacerein (0.81 ± 0.01) groups were significantly up-regulated compared with the PCOSgroup but still significantly downregulated compared with control (*P* < 0.05). The SIRT1 levels in PCOS+Myo-inositol were significantly downregulated (*P* < 0.05) compared with that of PCOS+Diacerein group

**Fig. 10:**
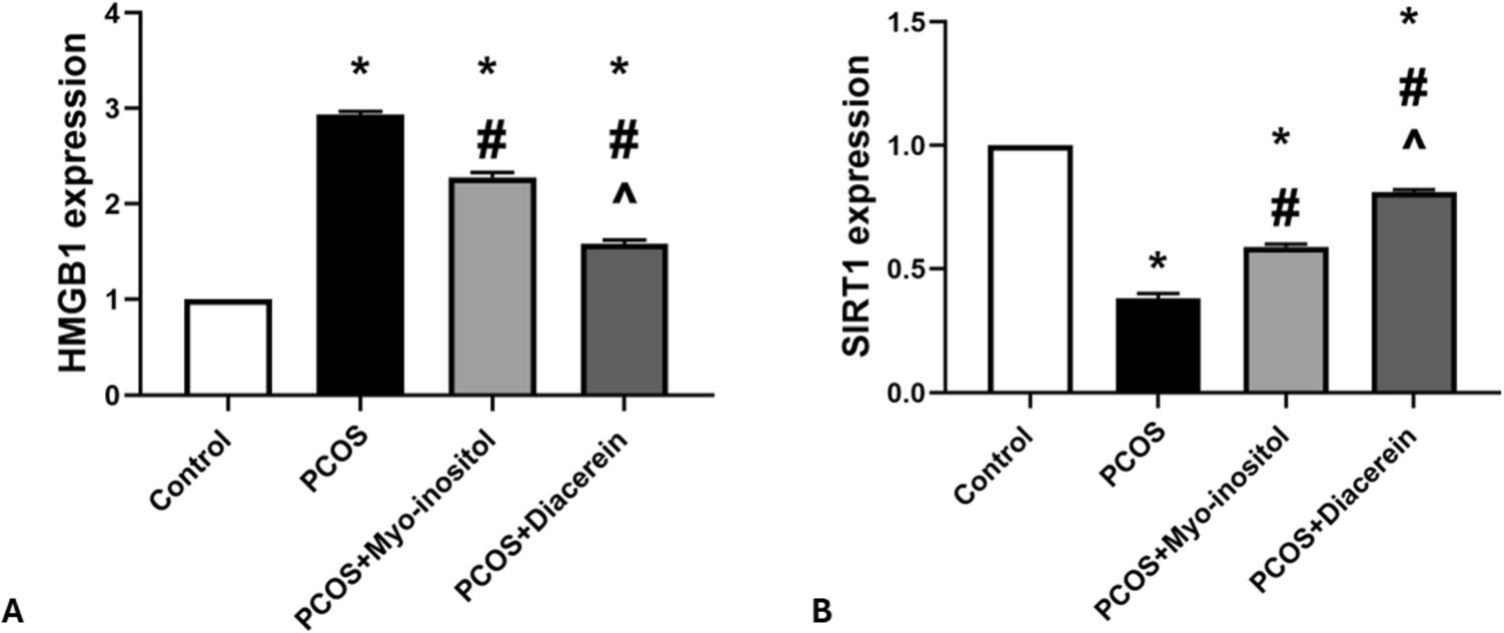
Effects of Myo-inositol and diacerein on SIRT1 and HMGB1 gene expression**. A** HMGB1 and **B** SIRT1. Compared using the ANOVA test; *p < 0.05 compared with control, #*p* < 0.05 compared with PCOS, and ^ compared with PCOS+Myo-inositol. PCOS, polycystic ovary syndrome; SIRT1, Sirtuin 1; HMGB1, high-mobility group box 1

The expression level of HMGB1 gene was significantly upregulated in PCOS (2.94 ± 0.03) compared with control group (1) (*P* < 0.05). HMGB1 levels in PCOS+Myo-inositol (2.28 ± 0.05) and PCOS+Diacerein (1.58 ± 0.04) groups were significantly downregulated compared with that in PCOS group but still significantly higher than that in control (*P* < 0.05). The HMGB1 levels in PCOS+Myo-inositol were significantly upregulated (*P* < 0.05) compared with that of PCOS + Diacerein (Fig. 10A, B).

### Histopathological results

#### Histopathological evaluation of the ovary

**Fig. 11:**
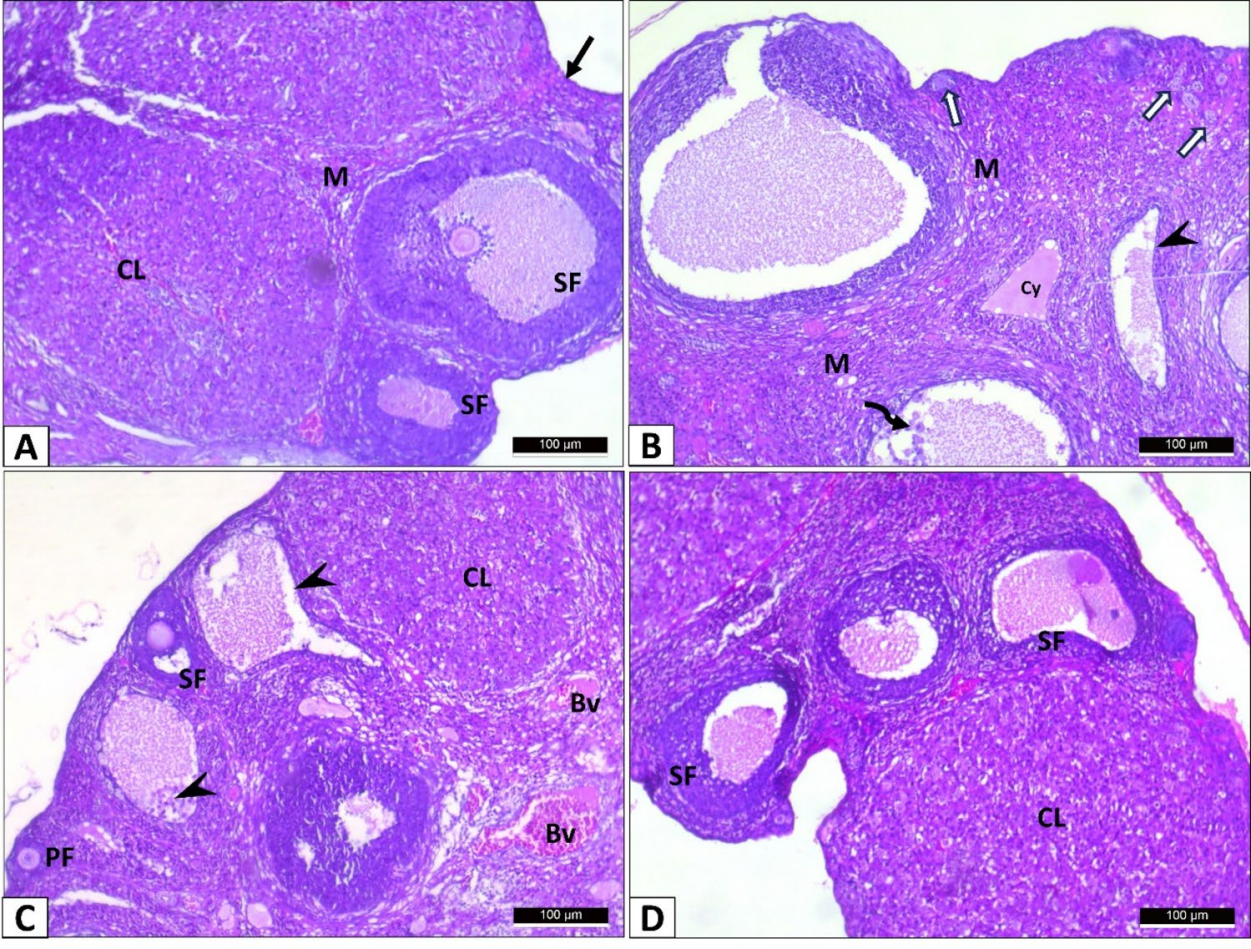
Representative H& E images of different experimental groups showing: **A** the control ovary rendering the normal histological picture; an outer cortex (arrow) and inner medulla (M). Secondary follicles at various stages of development (SF) and corpus luteum (CL) are detected. **B** PCOS group shows cystic follicle (cy), the follicle displays thin theca granulosa (arrowhead), and lumen containing cell debris, and other shows desquamation of the membrana granulosa cells (spiral arrows). Several primordial follicles (arrows) and vacuolated medulla (M) could be detected. **C** PCOS+Myo-inositol group showing primary follicle (PF), secondary follicle (SF), cystic follicles (arrowhead), large corpora lutea (CL), and dilated medullary blood vessel (Bv). **D** PCOS+Diacerein group showing secondary follicles (SF) and corpus luteum (CL) (H and E Χ100, Scale bar, 100 µm)

In the histological assessment of H&E-stained sections, the control rat ovary displayed several healthy follicles showing different stages of differentiation and large corpora lutea (Fig. 11A). Compared with the control rats, the PCOS group revealed fewer healthy follicles, several primordial follicles, and cystic follicles; follicle exhibits cell debris in the lumen with thin theca granulosa, and others showed desquamation of the membrana granulosa cells (Fig. 11B). Ovarian section of PCOS+Myo-inositol was composed of cortex and medulla, with big corpus luteum, and cystic follicles. The cortex revealed numerous primary and secondary follicles with some congested blood vessels (Fig. 11C). Ovary of PCOS+Diacerein showed corpus luteum and secondary follicles with few areas of congestion (Fig. 11D).

**Fig. 12:**
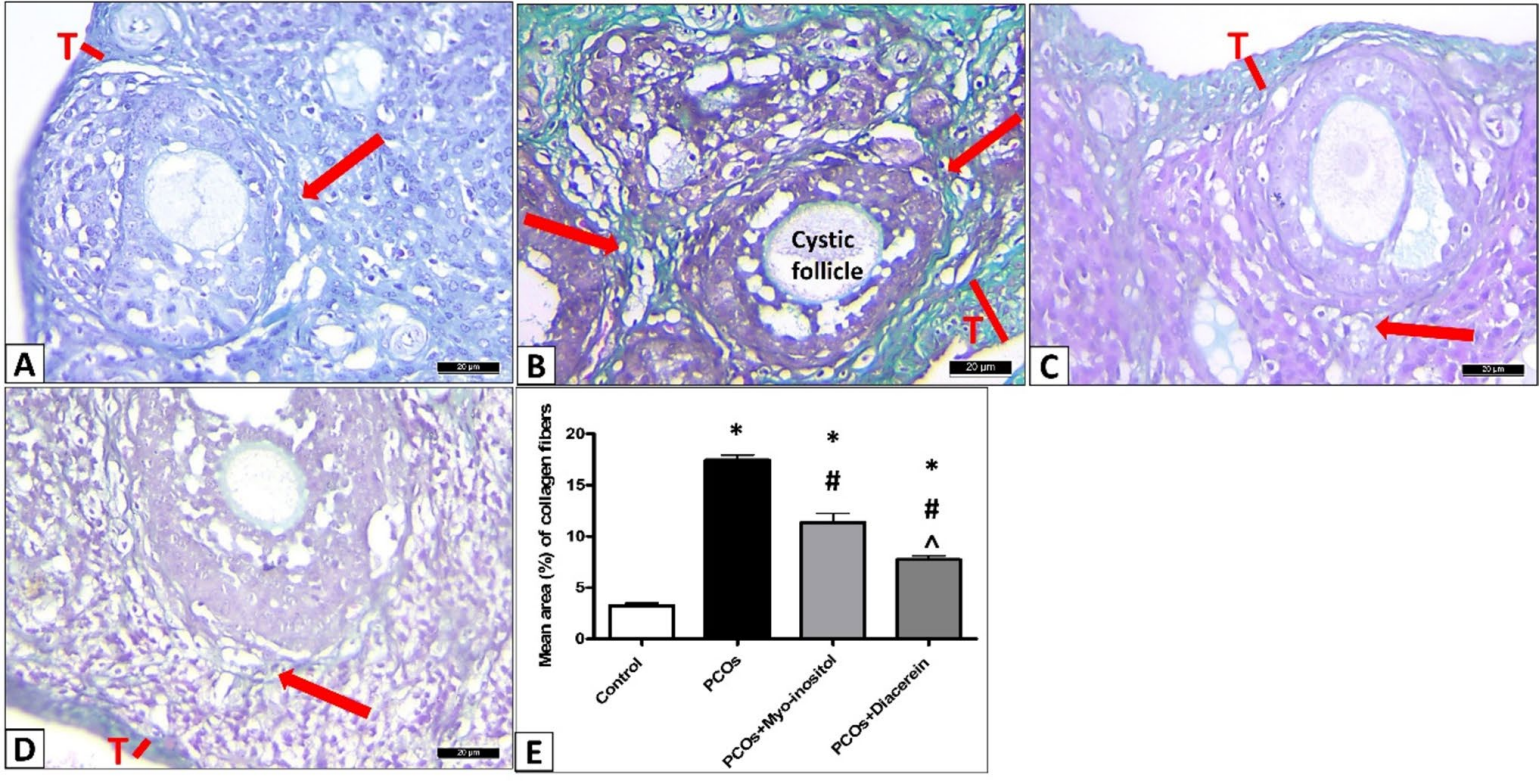
Representative photomicrographs of staining with Masson trichrome in the ovaries of all experimental groups. In the control group, collagen fibers are noticed in tunica albuginea (T) and around follicles (arrow). PCOS group reveals a significant increase in collagen fiber deposition in tunica albuginea (T) and around follicles (arrow) compared with control. These increases are significantly reduced in PCOS+Myo-inositol and PCOS+Diacerein groups. * Significant compared with control; # significant compared with PCOS, and ˄ significant compared with the PCOS+Myo-inositol. **A** control, **B** PCOS, **C** PCOS+Myo-inositol, **D** PCOS+Diacerein, **E** statistical analysis (Masson trichrome stain Χ400, Scale bar, 20 µm)

In the control group, Masson’s trichrome stain revealed that collagen was deposited in the tunica albuginea and the theca layer around the follicles (Fig. 12A). Compared with the control rats, a significant increment (17.46 ±1.19 vs. 3.233±0.62; *P* < 0.001) in the mean area percentage of deposited collagen in PCOS group was detected (Fig. 12B,E). A significant decrease in the mean area percentage of deposited collagen in the PCOS+Myo-inositol and PCOS+Diacerein compared with PCOS (11.35 ±2.19 and 7.745 ±0.89 respectively vs 17.46 ±1.19; *P* < 0.001). Collagen deposition diminished significantly in PCOS+Diacerein compared with PCOS+Myo-inositol (*P* < 0.001) despite being higher than that of the control rats (*P* < 0.001) (Fig. 12C,D,E).

#### Immunohistochemical assessment of NF-κB

**Fig. 13:**
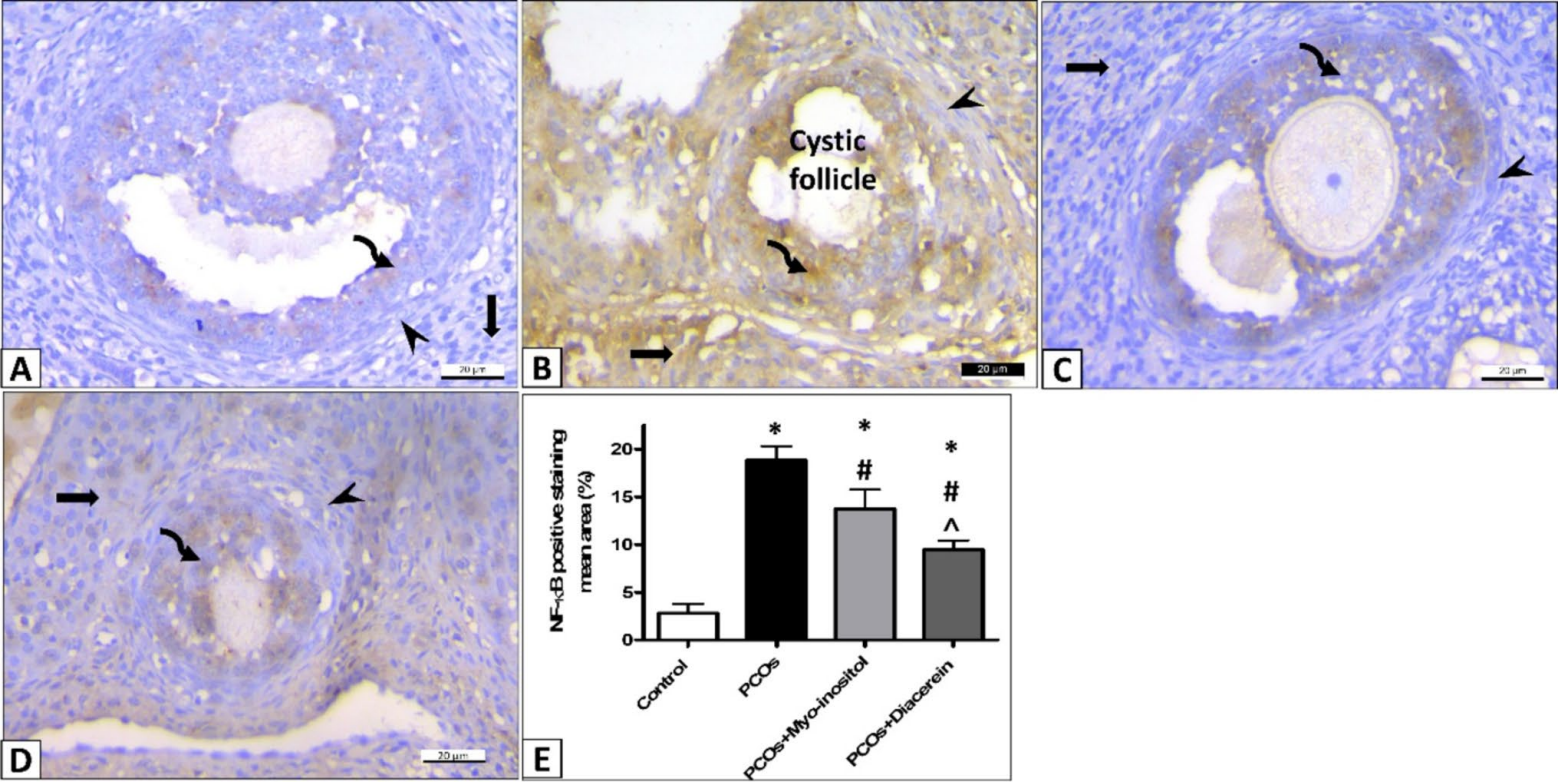
Representative photomicrographs of NF-κB expression in ovaries of all experimental groups. NF-κB expression in granulosa (curved arrow), theca cells (arrowhead), and interstitial cells (thick arrows) is dramatically upregulated in the PCOS compared with control. These increases are significantly downregulated in PCOS+Myo-inositol and PCOS+Diacerein. *Significant compared with control, # significant compared with PCOS, and ˄ significant compared with PCOS+Myo-inositol. **A** control, **B** PCOS, **C** PCOS+Myo-inositol, **D** PCOS+Diacerein, **E** statistical analysis (NF-κB Χ400, scale bar, 20 µm)

The area percentage of the NF-κB immunoreaction showed significant increment in the PCOS compared with the control values (18.83±1.47 vs 2.817±0.98; *P* < 0.001). But, the NF-κB immunoreaction was significantly downregulated in the PCOS+Myo-inositol and PCOS+Diacerein compared with PCOS values (13.76± 2.00 and 9.472± 0.97 vs 18.83±1.47; *P* < 0:001). PCOS+Diacerein revealed significant downregulation of immunoreaction than the PCOS+Myo-inositol (*P* < 0.001). However, values of both groups were significantly higher than control (*P* < 0.001) (Fig. 13).

#### Histopathological evaluation of the uterus

**Fig. 14:**
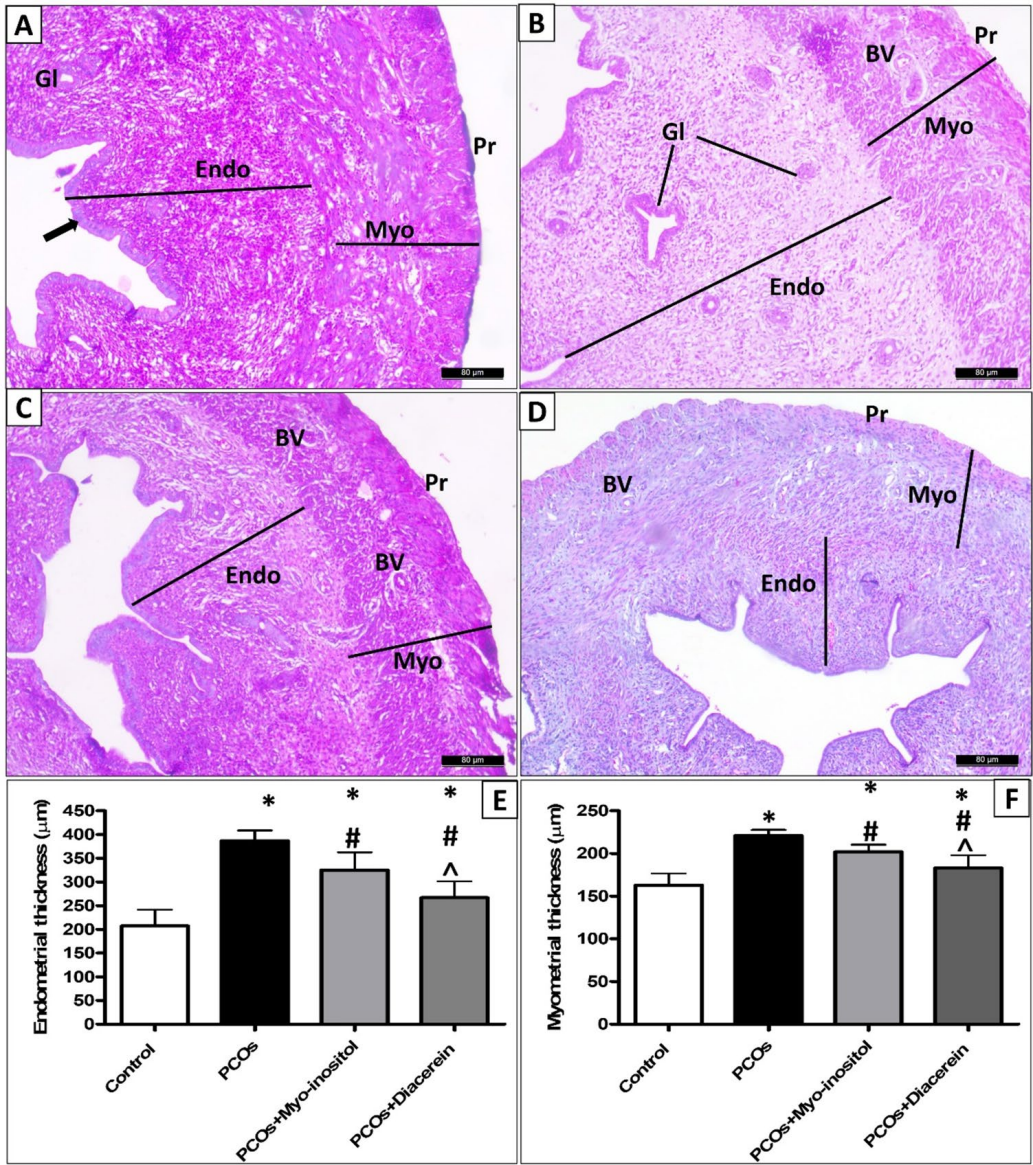
Representative H & E images of the uterus of different experimental groups showing the following: **A** the normal uterine architecture presented with simple columnar epithelial lining (arrow) with different layers; endometrium (Endo) with endometrial glands (Gl), myometrium (Myo) with normal thickness (line), and perimetrium (pr). **B** PCOS rats exhibiting marked endometrial hyperplasia (Endo) with an increased number of glands in the lamina propria (GL). The myometrial wall thickness is increased (line) with tiny blood vessels (Bv). **C, D** PCOS+Myo-inositol and PCOS+Diacerein respectively showing an apparent decrease in both endometrial (Endo) and myometrial wall (Myo) thickness (lines) with tiny blood vessels (Bv) in the myometrium. **E** Statistical analysis; * significant compared with control, # significant compared with PCOS, and **˄** significant compared with PCOS+Myo-inositol (H and E Χ200, Scale bar, 80 µm)

Normal architecture of the uterine histology in H&E-stained sections consisted of three layers: endometrium, myometrium, and perimetrium. The inner endometrium presented with intact folded and was lined by simple columnar epithelium. The lamina propria contain endometrial glands. The thick middle layer of the myometrium is constituted by layers of smooth muscles, and the outer perimetrium is presented with squamous epithelium (Fig. 14A). PCOS rats displayed the formation of multiple epithelial cell layers. In addition, the number of glands, endometrial wall, and myometrial wall thickness increased (386.9±21.73, 220.9±6.79 vs 207.0±34.26, and 162.9±13.54 respectively; *p*<0.001) (Fig. 14B, E, and F) compared with control. In contrast, PCOS+Myo-inositol and PCOS+Diacerein showed a decrease in both endometrial (325.4±37.19; *p*<0.05 and 267.0±34.22, *p*<0.001 respectively) and myometrial wall thickness (201.9±8.13;* p*<0.05 and 183.1±14.91, *p*<0.001 respectively) compared with PCOS (Fig. 14C, D, E, and F). Endometrial and myometrial thickness revealed a significant decrease (*p* < 0.05) in PCOS+Diacerein than PCOS+Myo-inositol, albeit both remained significantly higher than control (*p* < 0.05 and *p* < 0.001 respectively).

## Discussion

Letrozole, when administered continuously for 21 days, effectively induces PCOS-like features in rats (Balasubramanian et al. 2023). By inhibiting aromatase, letrozole reduces androgen conversion to estrogen, leading to excessive androgen accumulation in the ovaries. This hormonal imbalance disrupts follicle maturation and causes anovulation (Yang et al. 2021).

The letrozole-induced model of PCOS exhibits several features of PCOS in humans, like hyperglycemia, abnormal follicles, hyperandrogenism (Balasubramanian et al. 2023), and oxidative stress (Mirseyyed et al. 2023). Our findings align with previous findings, indicating that the induction of PCOS through letrozole administration resulted in elevated levels of LH and testosterone while simultaneously decreasing FSH levels compared with controls. Disruption of the normal hypothalamic-pituitary-gonadal axis increases both LH and testosterone levels in advanced disease (Doi et al. 2005; Marouf et al. 2022). Furthermore, diacerein and myoinositol administration rebalanced hormonal levels in the treated groups than in the PCOS group.

The H&E staining of the ovarian samples yielded results consistent with the preceding findings. As previously reported, PCOS ovaries showed the presence of numerous ovarian cysts as well as a deficiency of granulosa cell layers, oocytes, growing follicles, and corpus lutea (Arow et al. 2020; Ibrahim et al. 2022). The anovulatory state in PCOS, driven by elevated LH levels, led to the formation of antral follicles without ovulation, resulting in cystic follicles (Karimi Jashni et al. 2016). Conversely, the administration of myoinositol and diacerein successfully restored the typical structure of the ovaries, as demonstrated by the reduction in the number of cystic follicles and the increase in the numbers of corpus lutea, Graafian follicles, and developing primordial follicles. It has been previously reported that myoinositol increases oocyte quality during in vitro fertilization (Colazingari et al. 2013). Interestingly, these results were more pronounced with diacerein than the myoinositol-treated rats.

PCOS is a metabolic disorder characterized by an extensive interaction between insulin and the inflammatory signaling pathway. PCOS is characterized by hyperglycemia and IR (Singh et al. 2023). PCOS group in this study revealed significant hyperglycemia and IR, as indicated by elevated HOMA-IR. This finding aligns with prior studies that documented the induction of hyperglycemia in rats with PCOS induced by letrozole (Balasubramanian et al. 2023).

Myoinositol administration significantly improved glycemic state markers in PCOS rats. By competitively inhibiting duodenal glucose absorption, myoinositol helps reduce blood glucose levels (Di Nicolantonio and Hok 2022)***.*** Additionally, it plays a role in glucose transporter expression and cellular glucose uptake (Farias et al. 2019). Inositol metabolism has been found to be impaired in PCOS women (Merviel et al. 2021). Myoinositol functions as the secondary messenger in FSH signaling pathways within this particular tissue (Bizzarri et al. 2023). Multiple studies have confirmed the beneficial effects of myoinositol on hormonal and metabolic parameters in PCOS, leading to improved menstrual cycles and oocyte quality (Beresniak et al. 2023; Sharon et al 2024).

Remarkably, our results revealed that diacerein also decreased hyperglycemia and corrected IR. Li et al. documented that IL-1β specifically impairs the insulin-producing β-cells (in the pancreas) (Li et al. 2019). The administration of neutralizing monoclonal IL-1β antibodies for inhibiting IL-1β has resulted in improvements in hyperglycemia, insulin production, and HbA1c levels, as well as a reduction in inflammation in individuals with T2DM. These improvements can be attributed to improved β-cell function (Sloan-Lancaster et al. 2013). Our findings indicated that IL-1β was reduced in the diacerein-treated group than in the PCOS group. While myoinositol showed its antihyperglycemic effect by several metabolic mechanisms, diacerein exhibited comparable outcomes with slightly reduced effectiveness compared with myoinositol, which is likely attributed to its anti-inflammatory effect.

Dyslipidemia is a common complication of PCOS, often linked to hyperandrogenemia (Wahid et al. 2024)***.*** The present study showed a marked upregulation in serum cholesterol and TG in the PCOS group compared with controls. However, both myoinositol and diacerein-treated groups showed antihyperlipidemic effects, although myoinositol was slightly more effective. The superiority of myoinositol in ameliorating dyslipidemia could be attributed to its role in improving insulin sensitivity.

Oxidative stress contributes to PCOS pathogenesis. The deficiency in antioxidants such as GSH, catalase, and superoxide dismutase leads to the excessive generation of reactive oxygen species (ROS). Elevated levels of oxidants might influence steroidogenesis in the ovaries, ultimately leading to androgen production (Gao et al. 2023), which might impact the physiological function of ovaries, including oocyte maturation, folliculogenesis, and ovulation (Kafali et al. 2004).

In this study, PCOS rats had higher oxidative stress marker MDA with a marked reduction in the antioxidant defense of GSH compared with controls. MDA is a byproduct of lipid peroxidation, which triggers free radical damage to the cell membrane’s fatty acids, which causes cell necrosis and inflammation (Gao et al. 2023).

In the present study, diacerein showed its antioxidant effect by significantly reducing serum MDA levels and increasing GSH levels than in the PCOS group. Myoinositol showed similar results with moderately lower efficacy than diacerein. The ability of myoinositol to attenuate ROS production was reported previously (Osman et al. 2023).

Ibrahim et al. discovered a new mechanism for diacerein’s antihyperglycemic and insulin-sensitizing effects in individuals with PCOS. This is achieved through its ability to regulate antioxidants and potentially enhance glucose uptake in the liver as well as skeletal muscle, possibly through insulin-mediated mechanisms (Ibrahim et al. 2022). Furthermore, the antioxidant properties of diacerein were detected in different pathological models of rats, such as acute kidney and testicular injuries (Abdel-Gaber et al. 2018; Barakat et al. 2021).

Consistent with previous reports, our results revealed that PCOS rats exhibited increased TNF-α and IL-1β serum levels together with increased expression of NF-κB in ovarian tissue compared with the control group. NF-kB is recognized for its pivotal role in the control of immune responses and inflammation. During stress conditions, NF-kB translocates the nucleus and performs a transcriptional function in the generation of different proinflammatory cytokines, including TNF-α and IL-1β (Singh and Singh 2020).

In this study, myoinositol and diacerein-treated groups exhibited a significant decline in TNF-α and IL-1β serum levels, which probably rendered to sustaining NF-kB in an inactive state, proved by deceased its expression in the ovarian tissues using immunohistochemical assessment. Collectively, this demonstrates the anti-inflammatory and cytoprotective properties of these drugs, with diacerein exhibiting a more pronounced effect compared with myoinositol.

In this study, letrozole decreased the ovarian SIRT1 gene’s expression level compared with controls. SIRT1 exerts anti-inflammatory effects as it enhances macrophage polarization into the M2 phenotype with its ability to suppress inflammation (Yang et al. 2022)**.** Consistent with our results, Wu et al. detected that SIRT1 could inhibit NF-kB, so its expression is increased when SIRT1 is downregulated by the effect of letrozole (Wu et al. 2022). In addition, SIRT1 has been discovered to have the capability of protecting against PCOS by diminishing the presence of oxidative stress markers and methylglyoxal, which is closely associated with glycosylation stress, while also enhancing mitochondrial disorders (Wu et al. 2022). Moreover, SIRT1 has a positive effect on the regulation of insulin secretion in pancreatic β-cells and enhances insulin sensitivity, particularly in cases of IR (Lv et al. 2021).

Our results denote that diacerein- and myoinositol-treated groups showed increased SIRT-1 expression, associated with improved inflammation, oxidative stress, and IR. Myoinositol stimulates the adenosine monophosphate-activated protein kinase-SIRT1 signaling pathway, influencing adipocyte differentiation and the metabolism of fatty acids (Luo et al. 2022)***.*** Rhein, a major bioactive metabolite of diacerein, could have antidiabetic effects by antioxidant, anti-inflammatory properties and increasing the SIRT1 expression (Deng et al. 2022).

HMGB1, a damage-associated molecular pattern, was found to be related to IR (Zhang et al. 2020a). Consistent with prior finding, our study observed heightened HMGB1 expression in the ovaries of the PCOS group compared with controls, along with increased expression of NF-kB. When HMGB1 binds to receptors, it triggers the NF-kB signaling pathway and can potentially enhance the production of proinflammatory cytokines (Hu et al. 2023).

The myoinositol-treated group and diacerein-treated group showed downregulation of HMGB1 associated with decreased expression of the NF-kB, as proved by immunohistochemical results. This finding agrees with Cirillo et al., who concluded that HMGB1 increases in PCOS and reduces following treatment with myoinositol combined with alpha-lipoic acid (Cirillo et al. 2020). Also, Kamel et al. concluded that diacerein possesses antioxidant, anti-apoptotic, and anti-inflammatory characteristics against acetaminophen hepatotoxicity. These effects are attributable to HMGB1/TLR4/NF-κB pathway inhibition, as well as PPAR-γ expression’s upregulation (Mohamed Kamel et al. 2022).

Our study revealed that PCOS group rats showed elevated systolic, diastolic, and mean blood pressure. This result is consistent with Jovic et al., who linked hyperandrogenic PCOS to vascular and cardiac alterations (Joksimovic Jovic et al. 2021). Increased intima-media thickness and reduced nitrite levels in PCOS rats may contribute to hypertension (Jabbour et al. 2020; Joksimovic Jovic et al. 2021). Additionally, dysregulation of the hypothalamic-pituitary-adrenal and sympathetic-adrenal medullary axes in PCOS can contribute to elevated blood pressure (Zangeneh 2017).

Our results revealed that diacerein and myoinositol-treated groups showed reduced systolic, diastolic, and mean blood pressure levels when compared with the PCOS group. This finding may be rendered to the reduction in oxidative stress, inflammation, and the androgen level, as well as the improvement of dyslipidemia. The hypotensive effect of myoinositol supplementation was previously recorded (Arefhosseini et al. 2023). Recent data has shown that diacerein efficiently enhanced vascular function in rats with diabetes by reducing inflammation while minimizing IR (He et al. 2021)***.***

In this study, we compared the myo-electrical activity of the uterine muscles and isolated uterine muscle reactivity in response to serial concentrations of OXY and Ach between PCOS and control group rats. The results revealed increased amplitude and frequency of uterine muscle contraction in PCOS rats compared with controls. These outcomes are consistent with the results of Sajadi et al. who illustrated that Ach and OXY increase cytosolic Ca^2+^ levels in myometrial cells, leading to augmented uterine contractions (Sajadi et al. 2018).

Hyperandrogenism is associated with rise in myometrium area and thickness (Yusuf et al. 2023)***.*** Consistent with these findings, we noted a substantial rise in myometrium thickness in the PCOS group compared with controls. The enlarged myometrial thickness may be attributable to smooth muscle cell’s proliferation and/or the quantity of extracellular matrix components (Gomes et al. 2013). Furthermore, there may be a correlation between the thickening of the myometrium and alterations in uterine contractions in PCOS (Hosseinzadeh et al. 2021).

Nevertheless, the administration of myoinositol and diacerein resulted in a substantial reduction in the amplitude and frequency of uterine muscle contractions in rats with PCOS. This effect may be attributed to a decrease in androgen levels, as well as a reduction in endometrial and myometrial wall thickness. Diacerein’s anti-inflammatory properties may also contribute to these effects. In their study, Shi et al. found that diacerein could relieve bronchospasm smooth muscle hyperplasia and control airway inflammation in asthmatic mice by reducing several inflammatory factors (Shi et al. 2019).

In the current study, Masson’s trichrome stain revealed a significant increase in collagen fiber deposition in the ovarian tissues of PCOS group. This result was in line with a previous study (Nofal et al. 2019). Thickening of the theca cell layer, and an increased amount of collagen accumulated around follicles, might mechanically inhibit follicle rupture and ovulation (Zhang et al. 2013). Fibrosis might be due to chronic persistent inflammation (Krishna et al. 2017)**.**

Treatment with myoinositol or diacerein significantly decreased collagen fiber deposition in the ovary, which can be attributed to their anti-inflammatory properties. Diacerein has been previously shown to have protective anti-apoptotic and anti-inflammatory properties via NLRP3/caspase-1/IL-1β modulation (Fouad et al. 2020). In addition, the antifibrotic effect of diacerein may be attributed to the observed decrease in gene expression of HMGB1.

While the rat model is a useful tool for researching PCOS-related pathophysiology, differences in reproductive biology and disease manifestation may limit the generalizability of the findings. Future studies could go deeper into these variances and include more models or clinical data to validate the findings in a human setting.

Randomized controlled trials in human PCOS patients are required to establish the efficacy and safety of diacerein and myo-inositol as possible therapies. Future studies should investigate the optimal dosage, duration of treatment, and potential side effects. Overall, although our study’s results are encouraging, further research is crucial to confirm the clinical significance of diacerein and myo-inositol in the treatment of PCOS

## Conclusion

Our results support the use of letrozole in rat models for PCO-related research. We investigated and compared the potential therapeutic effects of diacerein and myoinositol in a rat model of PCOS, focusing on the underlying molecular mechanisms. Our findings indicate that both diacerein and myoinositol exhibit anti-inflammatory, antioxidant, hypolipidemic properties and help normalize blood glucose levels. Additionally, they can alleviate hypertension and reduce uterine muscle activity—key factors linked to the harmful effects of PCOS. Furthermore, they were found to restore structural changes in the ovary and uterus. These therapeutic effects appear to be mediated through the modulation of HMGB1, SIRT1, and NF-kB pathways. Diacerein displayed superiority over myo-inositol in alleviating the endocrine and most of the metabolic disturbances associated with PCOS.

## Data Availability

No datasets were generated or analysed during the current study.

## Declarations

**Ethical approval**

Ethical permissions from the Research Ethical Committee at the Faculty of Medicine, Menoufia University, Egypt with IRB No 12/2023 BIO-6 were obtained.
